# Development of Deep Ensembles to Screen for Autism and Symptom Severity Using Retinal Photographs

**DOI:** 10.1001/jamanetworkopen.2023.47692

**Published:** 2023-12-15

**Authors:** Jae Han Kim, JaeSeong Hong, Hangnyoung Choi, Hyun Goo Kang, Sangchul Yoon, Jung Yeon Hwang, Yu Rang Park, Keun-Ah Cheon

**Affiliations:** 1 Yonsei University College of Medicine, Severance Hospital, Yonsei University Health System, Seoul, Republic of Korea; 2 Department of Biomedical Systems Informatics, Yonsei University College of Medicine, Seoul, Republic of Korea; 3 Department of Child and Adolescent Psychiatry, Severance Hospital, Yonsei University College of Medicine, Seoul, Republic of Korea; 4 Institute of Behavioral Science in Medicine, Yonsei University College of Medicine, Yonsei University Health System, Seoul, Republic of Korea; 5 Department of Ophthalmology, Institute of Vision Research, Severance Eye Hospital, Yonsei University College of Medicine, Seoul, Republic of Korea; 6 Department of Medical Humanities and Social Sciences, Yonsei University College of Medicine, Seoul, Republic of Korea

## Abstract

**Importance:**

Screening for autism spectrum disorder (ASD) is constrained by limited resources, particularly trained professionals to conduct evaluations. Individuals with ASD have structural retinal changes that potentially reflect brain alterations, including visual pathway abnormalities through embryonic and anatomic connections. Whether deep learning algorithms can aid in objective screening for ASD and symptom severity using retinal photographs is unknown.

**Objective:**

To develop deep ensemble models to differentiate between retinal photographs of individuals with ASD vs typical development (TD) and between individuals with severe ASD vs mild to moderate ASD.

**Design, Setting, and Participants:**

This diagnostic study was conducted at a single tertiary-care hospital (Severance Hospital, Yonsei University College of Medicine) in Seoul, Republic of Korea. Retinal photographs of individuals with ASD were prospectively collected between April and October 2022, and those of age- and sex-matched individuals with TD were retrospectively collected between December 2007 and February 2023. Deep ensembles of 5 models were built with 10-fold cross-validation using the pretrained ResNeXt-50 (32×4d) network. Score-weighted visual explanations for convolutional neural networks, with a progressive erasing technique, were used for model visualization and quantitative validation. Data analysis was performed between December 2022 and October 2023.

**Exposures:**

Autism Diagnostic Observation Schedule–Second Edition calibrated severity scores (cutoff of 8) and Social Responsiveness Scale–Second Edition T scores (cutoff of 76) were used to assess symptom severity.

**Main Outcomes and Measures:**

The main outcomes were participant-level area under the receiver operating characteristic curve (AUROC), sensitivity, and specificity. The 95% CI was estimated through the bootstrapping method with 1000 resamples.

**Results:**

This study included 1890 eyes of 958 participants. The ASD and TD groups each included 479 participants (945 eyes), had a mean (SD) age of 7.8 (3.2) years, and comprised mostly boys (392 [81.8%]). For ASD screening, the models had a mean AUROC, sensitivity, and specificity of 1.00 (95% CI, 1.00-1.00) on the test set. These models retained a mean AUROC of 1.00 using only 10% of the image containing the optic disc. For symptom severity screening, the models had a mean AUROC of 0.74 (95% CI, 0.67-0.80), sensitivity of 0.58 (95% CI, 0.49-0.66), and specificity of 0.74 (95% CI, 0.67-0.82) on the test set.

**Conclusions and Relevance:**

These findings suggest that retinal photographs may be a viable objective screening tool for ASD and possibly for symptom severity. Retinal photograph use may speed the ASD screening process, which may help improve accessibility to specialized child psychiatry assessments currently strained by limited resources.

## Key Points

**Question:**

Can deep learning models screen individuals for autism spectrum disorder (ASD) and symptom severity using retinal photographs?

**Findings:**

In this diagnostic study of 1890 eyes of 958 participants, deep learning models had a mean area under the receiver operating characteristic curve of 1.00 for ASD screening and 0.74 for symptom severity. The optic disc area was also important in screening for ASD.

**Meaning:**

These findings support the potential of artificial intelligence as an objective tool in screening for ASD and possibly for symptom severity using retinal photographs.

## Teaser

This diagnostic study examines the potential of deep ensemble models to screen for autism spectrum disorder and symptom severity using retinal photographs.

## Introduction

Autism spectrum disorder (ASD) is characterized by 2 core areas of symptoms: social communication impairment and restricted and repetitive behaviors or interests.^1^ In 2020, the US Centers for Disease Control and Prevention estimated that the prevalence of ASD was 1 in 36; this number continues to grow, possibly due to increased awareness among the public, medical, and research communities.^2,3^ The 2019 Global Burden of Disease study reported an age-standardized global ASD prevalence of 369.39 per 100 000.^4^ Several ASD screening tools have demonstrated notable performance.^5,6^ For example, the Modified Checklist for Autism in Toddlers is primarily based on caregiver report, with sensitivity of 0.83 (95% CI, 0.77-0.88) and specificity of 0.94 (95% CI, 0.89-0.97)^7^; however, caregiver assessment is influenced by one’s understanding of the child’s developmental milestones.^8^ The Social Attention and Communication Surveillance is conducted by trained professionals, and it exhibits sensitivity of 0.96 (95% CI, 0.94-0.98) and specificity of 1.00 (95% CI, 0.99-1.00) for ages 12 to 42 months.^9^ The growing demands for ASD evaluation cannot be met with limited resources, including trained specialists.^10^ In addition, because of the substantial time required to evaluate individuals suspected of having ASD, inaccessibility to medical services has increased.^11^ Therefore, objective ASD screening methods are increasingly needed.

Retinal photographs have been proposed as a potential objective screening tool for ASD, with the theoretical background that the retina may be used to indirectly assess structural abnormalities of the brain.^12,13^ Accordingly, retinal alterations in individuals with ASD have been observed compared with individuals with typical development (TD).^14,15,16,17,18^ In a previous study, machine learning models were developed to screen for ASD using retinal photographs, with reported sensitivity of 0.96 (95% CI, 0.76-1.00) and specificity of 0.91 (95% CI, 0.71-0.99).^19^ Nevertheless, these results were difficult to generalize owing to the small number of participants. To our knowledge, there have been no attempts to screen for ASD symptom severity using artificial intelligence despite the observed association between retinal alterations and symptom severity.^14,15^

Although there are promising results with deep neural networks in various domains, they tend to lack the ability to quantify predictive uncertainty and produce overconfident predictions.^20^ Thus, we used deep ensembles to robustly estimate uncertainty and improve predictive performance.^20,21^ Accordingly, this study aimed to investigate whether retinal photographs can serve as an objective screening tool for ASD and symptom severity by generating deep ensemble models with a larger number of participants. Furthermore, we tested their possible use in a pediatric population via sequential age-based modeling.

## Methods

This diagnostic study was approved by the Institutional Review Board of Severance Hospital, Yonsei University, Seoul, Republic of Korea. Written informed consent was obtained from all participants with ASD. The consent requirement for individuals with TD was waived because retrospective and deidentified data were used. The study followed the Standards for Reporting of Diagnostic Accuracy Studies (STARD) reporting guideline.

### Participant Recruitment and Study Variables

Children and adolescents (aged <19 years) with ASD were recruited from the Department of Child and Adolescent Psychiatry, Severance Hospital, Yonsei University College of Medicine, between April and October 2022. Retinal photographs of age- and sex-matched control participants with TD were retrospectively collected at the Department of Ophthalmology, Severance Hospital, Yonsei University College of Medicine, between December 2007 and February 2023. A detailed description of participant recruitment and the retinal imaging environment is provided in eMethods 1 in Supplement 1.

We excluded individuals with ASD and a diagnosed major psychiatric disorder (eg, bipolar disorder or conditions within the schizophrenia spectrum and other psychotic disorders), individuals with TD and any psychiatric disorder (eg, ASD, attention-deficit/hyperactivity disorder, bipolar disorder, or schizophrenia spectrum and other psychotic disorders), and individuals with a neurologic illness (eg, epilepsy, encephalitis, demyelinating disease, or traumatic brain injury) or eye disease that may affect the retinal fundus (eg, glaucoma, retinopathy of prematurity, or ophthalmic surgical history). A participant flow diagram is presented in eFigure 1 in Supplement 1.

Symptom severity was assessed using Autism Diagnostic Observation Schedule–Second Edition (ADOS-2) calibrated severity scores and Social Responsiveness Scale–Second Edition (SRS-2) T scores. The cutoff for symptom severity was established at 8 for the ADOS-2 and 76 for the SRS-2, according to previous publications.^22,23^ We evaluated the full-scale intelligence quotient (FSIQ) for individuals with ASD. Assessment with the ADOS-2 and FSIQ is detailed in eMethods 2 in Supplement 1.

### Data Preprocessing

Retinal photographs were preprocessed by removing the noninformative area outside the fundus circle and resizing the image to 224 × 224 pixels. When we generated the ASD screening models, we cropped 10% of the image top and bottom before resizing because most images from participants with TD had noninformative artifacts (eg, panels for age, sex, and examination date) in 10% of the top and bottom.

### Model Development

Deep ensembles offer advantages over single models because they exhibit superior performance and a greater capacity to quantify predictive uncertainty.^21^ We used convolutional neural networks with the ResNeXt-50 (32×4d) network as the backbone to construct classification ensemble models to screen for ASD (ie, ASD vs TD) and ASD symptom severity (ie, severe vs mild to moderate symptoms) using retinal photographs.^24^ The training process is described in eMethods 3 in Supplement 1. To screen for ASD, we examined 2 settings: (1) differentiation between TD and ASD diagnosed solely with *Diagnostic and Statistical Manual of Mental Disorders, Fifth Edition* (*DSM-5*) criteria^1^ and (2) differentiation between TD and ASD diagnosed with the *DSM-5* criteria and ADOS-2 scores. For ASD symptom severity screening, we investigated 2 measurements: ADOS-2 calibrated severity scores (≥8 vs <8) and SRS-2 T scores (≥76 vs <76).^22,23^

The data sets were randomly divided into training (85%) and test (15%) sets. We used 10-fold cross-validation to obtain generalized results of model performance. Data splitting was performed at the participant level and stratified based on the outcome variables. Because the data classes were imbalanced for symptom severity (ADOS-2 and SRS-2), we performed a random undersampling of the data at the participant level before conducting data splitting. Moreover, we examined different split ratios (80:20 and 90:10) to assess the robustness and consistency of the predictive performances across diverse splitting proportions.

To assess the feasibility of our approach in a pediatric population, we performed sequential age-based modeling to screen for ASD. We initially built models using images from the youngest age group within our sample, starting at age 4 years (ie, the minimum age group in our data set). Subsequently, we repeated this process, developing models for participants aged 5 years or younger, then those aged 6 years or younger, and so on.

### Uncertainty Estimation

We used a publicly available data set for uncertainty estimation as an out-of-distribution set containing 1000 retinal photographs of 39 different fundus diseases.^25^ We excluded the 38 images of healthy individuals without fundus disease because our data set contained images from individuals with TD, resulting in the utilization of 962 images.

We calculated entropy to estimate the predictive uncertainty for each image in the test and out-of-distribution sets. Entropy ranged from 0 to 1, with a larger value indicating a higher predictive uncertainty. We hypothesized that entropies in the out-of-distribution data would be larger than those in the test set because our models trained on images of individuals with ASD or TD would yield higher uncertainty for out-of-distribution data.

### Model Visualization and Quantitative Validation

We used score-weighted visual explanations for convolutional neural networks to explore the areas deemed important for making predictions.^26^ The target layer was the final convolutional layer before the pooling layer in ResNeXt-50 (32×4d). Because we constructed 5 models to create deep ensembles, we averaged the 5 heat maps for each image. Subsequently, we used a progressive erasing technique to quantitatively validate the explainability of the heat map.^27,28^ Model performance was sequentially evaluated by gradually removing 5% of the least important parts based on the averaged heat map. The eliminated parts of the image were filled with zeros, which were equivalent to black.

### Statistical Analysis

Differences in clinical variables between the 2 groups were assessed using independent *t* tests or χ^2^ tests. Mann-Whitney *U* tests were used to compare entropies between the test and out-of-distribution sets. Additionally, classification performance was evaluated based on the area under the receiver operating characteristic curve (AUROC), sensitivity, specificity, and accuracy.^29^ Calibration performance was measured using the negative log-likelihood (NLL) and Brier score.^30,31^ Model performance metrics were calculated at the participant level. We estimated the 95% CI for each estimate using the bootstrapping method with 1000 resamples.

All statistical tests were 2 sided, and statistical significance was set at *P* < .05. All statistical analyses were performed using Python, version 3.9.7 (Python Software Foundation), and all classification models were implemented using PyTorch, version 1.12.0 (PyTorch Foundation). Data analysis was performed between December 2022 and October 2023.

## Results

### Study Dataset

This study included 1890 eyes of 958 participants. The ASD and TD groups each included 479 participants (945 eyes), had a mean (SD) age of 7.8 (3.2) years, and comprised more boys (392 [81.8%]) than girls (87 [18.2%]). Of the 479 participants with ASD, 436 (91.0%) had an FSIQ reported (mean [SD], 70.2 [20.5]). An ADOS-2 calibrated severity score was reported for 241 participants (50.3%) with ASD (mean [SD], 7.0 [1.3]). All 479 participants (100%) with ASD had an SRS-2 T score available (mean [SD], 86.2 [17.7]). Participant characteristics are summarized in Table 1.

**Table 1.** Participant Characteristics

Characteristic	Participants with ASD (n = 479 with 945 images)	Participants with TD (n = 479 with 945 images)	*P* value
Age, mean (SD), y	7.8 (3.2)	7.8 (3.2)	>.99
Sex, No. (%)			
Male	392 (81.8)	392 (81.8)	>.99
Female	87 (18.2)	87 (18.2)	
FSIQ			
No. (%)	436 (91.0)	NA	NA
Mean (SD)	70.2 (20.5)	NA	
Symptom severity			
ADOS-2 calibrated severity score			
No. (%)	241 (50.3)	NA	NA
Mean (SD)	7.0 (1.3)	NA	
SRS-2 T score			
No. (%)	479 (100)	NA	NA
Mean (SD)	86.2 (17.7)	NA	

Abbreviations: ADOS-2, Autism Diagnostic Observation Schedule–Second Edition; ASD, autism spectrum disorder; FSIQ, full-scale intelligence quotient; NA, not available; SRS-2, Social Responsiveness Scale–Second Edition; TD, typical development.

### Model Performance for ASD Screening

To differentiate between TD and ASD diagnosed solely with the *DSM-5* criteria, 1890 retinal photographs (945 each for TD and ASD) were included. The 10 models had a mean AUROC, sensitivity, specificity, and accuracy of 1.00 (95% CI, 1.00-1.00) for the test set. These models had successful calibration performance, as indicated by a mean NLL of 0 and a mean Brier score of 0. Classification and calibration performances were retained even when limited to ASD diagnosis using *DSM-5* criteria and ADOS-2 scores (Table 2). Notably, model performance was retained regardless of the split ratio (eTable 1 in Supplement 1). Furthermore, our sequential age-based modeling suggested that the predictive performance was retained in all age groups, even for those aged 4 years (eTable 2 in Supplement 1).

**Table 2.** Mean Performance of Cross-Validated Single Models and Deep Ensembles to Screen for ASD and Symptom Severity

	Mean classification performance (95% CI)				Mean calibration performance (95% CI)	
	AUROC	Sensitivity	Specificity	Accuracy	NLL	Brier score
ASD vs TD^a^						
Single model	1.00 (1.00-1.00)	1.00 (1.00-1.00)	1.00 (1.00-1.00)	1.00 (1.00-1.00)	0	0
Deep ensemble	1.00 (1.00-1.00)	1.00 (1.00-1.00)	1.00 (1.00-1.00)	1.00 (1.00-1.00)	0	0
ASD vs TD^b^						
Single model	1.00 (1.00-1.00)	1.00 (1.00-1.00)	1.00 (1.00-1.00)	0.99 (0.99-1.00)	0.02 (0.00-0.05)	0.001 (0.000-0.001)
Deep ensemble	1.00 (1.00-1.00)	1.00 (1.00-1.00)	1.00 (1.00-1.00)	1.00 (1.00-1.00)	0	0
ADOS-2 calibrated severity score (≥8 vs <8)						
Single model	0.67 (0.64-0.70)	0.58 (0.54-0.62)	0.67 (0.63-0.71)	0.62 (0.60-0.65)	12.99 (12.09-13.93)	0.38 (0.35-0.40)
Deep ensemble	0.74 (0.67-0.80)	0.58 (0.49-0.66)	0.74 (0.67-0.82)	0.66 (0.60-0.73)	11.76 (9.50-13.82)	0.34 (0.28-0.40)
SRS-2 T score (≥76 vs <76)						
Single model	0.46 (0.43-0.48)	0.50 (0.47-0.53)	0.45 (0.42-0.48)	0.47 (0.45-0.50)	18.17 (17.45-18.96)	0.53 (0.51-0.55)
Deep ensemble	0.44 (0.38-0.50)	0.52 (0.46-0.59)	0.44 (0.38-0.51)	0.48 (0.44-0.53)	17.83 (16.31-19.44)	0.52 (0.47-0.56)

Abbreviations: ADOS-2, Autism Diagnostic Observation Schedule–Second Edition; ASD, autism spectrum disorder; AUROC, area under the receiver operating characteristic curve; NLL, negative log-likelihood; SRS-2, Social Responsiveness Scale–Second Edition; TD, typical development.

^a^ Diagnosis of ASD based on the *Diagnostic and Statistical Manual of Mental Disorders, Fifth Edition* criteria only.

^b^ Diagnosis of ASD based on both the *Diagnostic and Statistical Manual of Mental Disorders, Fifth Edition* criteria and ADOS-2 scores.

### Model Performance for ASD Symptom Severity Screening

To screen for symptom severity measured with ADOS-2 calibrated severity scores, 305 retinal photographs were used (154 for scores ≥8 and 151 for scores <8). The 10 models differentiated severe ASD from mild to moderate ASD measured with the ADOS-2 at the participant level, with a mean AUROC of 0.74 (95% CI, 0.67-0.80), sensitivity of 0.58 (95% CI, 0.49-0.66), specificity of 0.74 (95% CI, 0.67-0.82), and accuracy of 0.66 (95% CI, 0.60-0.73) for the test set. Regarding calibration performance, the deep ensemble models (mean NLL, 11.76 [95% CI, 9.50-13.82]; mean Brier score, 0.34 [95% CI, 0.28-0.40]) outperformed single models (Table 2). With the 80:20 split, the models had a mean AUROC of 0.71 (95% CI, 0.52-0.90); with the 90:10 split, the models had a mean AUROC of 0.79 (95% CI, 0.55-1.00; eTable 1 in Supplement 1).

To screen for symptom severity measured with SRS-2 scores, 556 retinal photographs were used (277 for scores ≥76 and 279 for scores <76). The models failed to screen for SRS-2–based symptom severity, with a mean AUROC of 0.44 (95% CI, 0.38-0.50), sensitivity of 0.52 (95% CI, 0.46-0.59), specificity of 0.44 (95% CI, 0.38-0.51), and accuracy of 0.48 (95% CI, 0.44-0.53) for the test set (Table 2). The classification failed in all split ratios (eTable 1 in Supplement 1). The receiver operating characteristic curves for both tasks are presented in eFigure 2 in Supplement 1.

### Uncertainty Estimation

**Figure 1.**
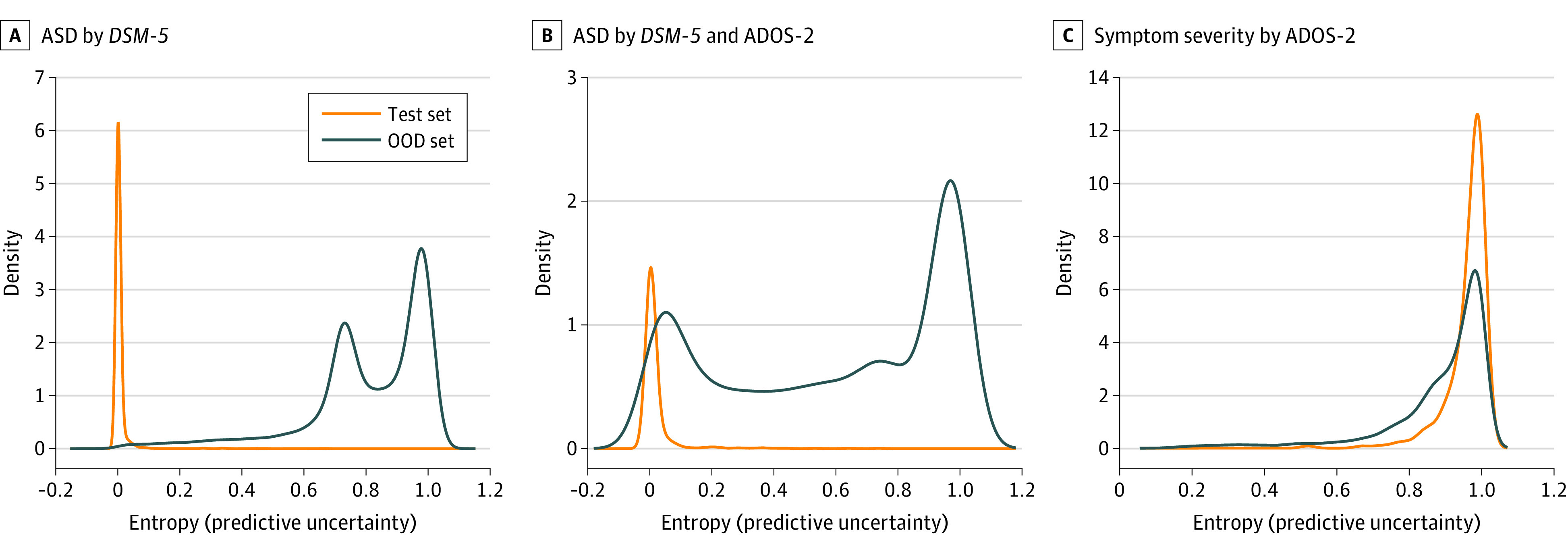
Density Plots of Predictive Uncertainty on the Test and Out-of-Distribution (OOD) Sets A to C, Screening for autism spectrum disorder (ASD) with the *Diagnostic and Statistical Manual of Mental Disorders, Fifth Edition* criteria only (A), for ASD with the *Diagnostic and Statistical Manual of Mental Disorders, Fifth Edition* criteria and Autism Diagnostic Observation Schedule–Second Edition scores (B), and for symptom severity based on Autism Diagnostic Observation Schedule–Second Edition scores (C).

The models used to screen for ASD diagnosed with the *DSM-5* criteria produced significantly lower entropies for the test set than for the out-of-distribution set (mean [SD], 0.01 [0.03] vs 0.8 [0.2]; *P* < .001; Figure 1A). This trend was also observed in the models used to screen for ASD diagnosed with the *DSM-5* criteria and ADOS-2 scores for the test set and the out-of-distribution set (mean [SD], 0.02 [0.06] vs 0.6 [0.4]; *P* < .001; Figure 1B). However, the models used to screen for symptom severity measured with ADOS-2 scores had high entropies for both the test set and the out-of-distribution set (mean [SD], 1.0 [0.06] vs 0.9 [0.2]) (Figure 1C).

### Model Visualization and Quantitative Validation

**Figure 2.**
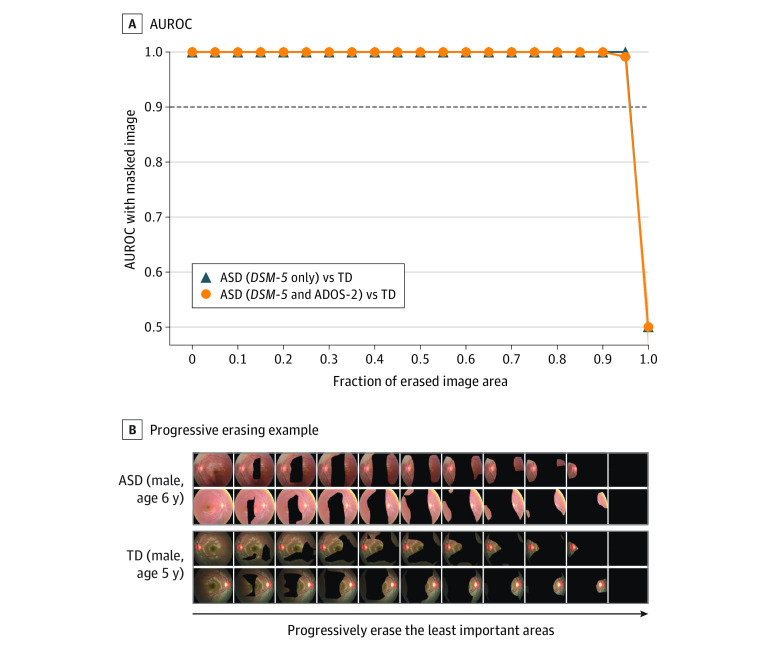
Quantitative Validation of the Heat Map With the Progressive Erasing Technique for Autism Spectrum Disorder (ASD) Screening A, Area under the receiver operating characteristic curve (AUROC) with shaded 95% CI obtained from masked images. B, Progressive erasing for ASD and typical development (TD). ADOS-2 indicates Autism Diagnostic Observation Schedule–Second Edition; *DSM-5*, *Diagnostic and Statistical Manual of Mental Disorders, Fifth Edition*.

**Figure 3.**
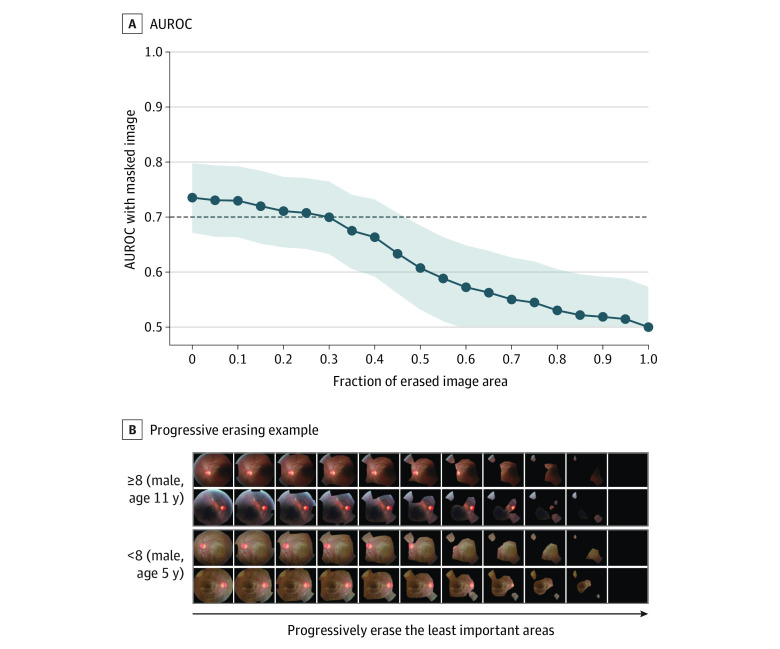
Quantitative Validation of the Heat Map With the Progressive Erasing Technique for ADOS-2–Based Symptom Severity Screening A, Area under the receiver operating characteristic curve (AUROC) with shaded 95% CI obtained from masked images. B, Progressive erasing for severe autism spectrum disorder (ASD) and mild to moderate ASD. ADOS-2 indicates Autism Diagnostic Observation Schedule–Second Edition.

Figure 2 presents quantitative validation of the heat maps for ASD screening. There was no notable decrease in the mean AUROC, even when 95% of the least important areas were removed, regardless of the diagnostic method. Heat maps highlighted the optic disc area. However, 70% of the image was needed to achieve a mean AUROC of 0.70 in screening for symptom severity using ADOS-2 scores (Figure 3).

## Discussion

The findings of this study suggest that retinal photographs may serve as a viable candidate for an objective method to screen for ASD and possibly for symptom severity. The mean AUROC values for ASD screening and symptom severity were 1.00 (95% CI, 1.00-1.00) and 0.74 (95% CI, 0.67-0.80), respectively. Our results also support that the optic disc area is an important region in ASD screening. Moreover, the models for screening ASD exhibited higher uncertainty for the out-of-distribution set than for the test set with excellent calibration performance, implying that they could robustly quantify predictive uncertainty.

Our models had promising performance in differentiating between ASD and TD using retinal photographs, implying that retinal alterations in ASD may have potential value as biomarkers. Interestingly, these models retained a mean AUROC of 1.00 using only 10% of the image containing the optic disc, indicating that this area is crucial for distinguishing ASD from TD. Considering that a positive correlation exists between retinal nerve fiber layer (RNFL) thickness and the optic disc area,^32,33^ previous studies that observed reduced RNFL thickness in ASD compared with TD^14,15,16^ support the notable role of the optic disc area in screening for ASD. Given that the retina can reflect structural brain alterations as they are embryonically and anatomically connected,^12^ this could be corroborated by evidence that brain abnormalities associated with visual pathways are observed in ASD. First, reduced cortical thickness of the occipital lobe was identified in ASD when adjusted for sex and intelligence quotient.^34^ Second, ASD was associated with slower development of fractional anisotropy in the sagittal stratum where the optic radiation passes through.^35^ Interestingly, structural and functional abnormalities of the visual cortex and retina have been observed in mice that carry mutations in ASD-associated genes, including *Fmr1*, *En2*, and BTBR,^36,37,38^ supporting the idea that retinal alterations in ASD have their origins at a low level. However, in clinical practice, the question extends beyond identifying ASD solely to also include ASD with attention-deficit/hyperactivity disorder, other psychiatric disorders, and their combination.^39,40,41^ Further studies that involve different neurodevelopmental or psychiatric disorders are needed to identify retinal alterations specific to each disorder and develop a multiclassification model.

Despite its promising performance, the applicability of our approach to the pediatric population remained a key concern given that the primary aim of ASD screening is early detection for timely intervention.^42,43^ Our sequential age-based modeling suggested that retinal photographs may serve as an objective screening tool starting at least at age 4 years. Moreover, the newborn retina continues to develop and mature up to age 4 years.^44,45^ Taken together, our models are potentially viable for screening children from this age onward, which is earlier than the average age of 60.48 months at ASD diagnosis.^46^ However, this does not indicate that retinal photographs are not feasible for individuals aged younger than 4 years. This question remains unexplored because the youngest age group in our sample was 4 years. Retinal alterations in individuals with ASD may manifest even before retinal maturation. Therefore, further research with participants aged younger than 4 years is essential.

Our findings suggest that retinal photographs may provide additional information regarding symptom severity. We observed that feasible classification was achievable only for ADOS-2 scores and not for SRS-2 scores. This may be because the ADOS-2 is conducted by a trained professional with ample time for assessment, whereas the SRS-2 is typically completed by a caregiver in a few dozen minutes; thus, the former would reflect one’s severity status more accurately than the latter. Proportional retinal alterations by symptom severity seemed robust given that the RNFL thickness in a previous study^14^ was thinner in the group with high-functioning ASD (mean ADOS score, 14.2) than in a group with Asperger syndrome (mean ADOS score, 10.4). Moreover, ASD with higher ADOS scores was associated with the slower development of fractional anisotropy in the sagittal stratum.^35^

### Limitations

This study had several limitations. First, we used a single-center data set, which may limit the generalizability of our findings. However, this allowed us to confirm the potential of retinal photographs as viable candidates for screening tools for ASD by controlling the expected variability owing to retinal photography settings.^47^ Future studies that use multicenter data sets would be beneficial. Second, retinal photographs may not be sufficient for screening symptom severity because they can only assess retinal alterations in a 2-dimensional space, whereas the retina is a 3-dimensional structure with multiple layers. Therefore, further studies using optical coherence tomography are warranted. Third, the medication status of participants with ASD, which could have affected the retina, was not fully controlled. However, there has been no corroborating evidence regarding the secondary changes in the RNFL or optic disc related to the toxicity of atypical antipsychotic medications.^48,49^ Future studies involving medication-naive patients with ASD are needed to investigate this relationship. Fourth, the exclusion of concurrent medical, neurological, and psychiatric conditions suggests that our models may apply to only a portion of individuals with ASD in clinical practice given that a substantial portion of ASD is associated with coexisting conditions.^39,40,41^ However, this approach allowed us to investigate the association between ASD and the retina while mitigating the potential influence of these conditions. Fifth, our models were limited to differentiating between individuals with ASD and TD; the primary challenge remains to distinguish ASD from a multitude of other neurodevelopmental or psychiatric disorders, which warrants further investigation.

## Conclusions

This diagnostic study examined the potential of deep learning algorithms to screen for ASD and possibly symptom severity using retinal photographs. Our findings suggest that the optic disc area is crucial for differentiating between individuals with ASD and TD. Although future studies are required to establish generalizability, our study represents a notable step toward developing objective screening tools for ASD, which may help address urgent issues such as the inaccessibility of specialized child psychiatry assessments due to limited resources.

## Notes

Supplement 1. **eMethods 1.** Detailed Description of the Retinal Imaging Environment **eFigure 1.** Participant Flow Diagram **eFigure 2.** Receiver Operating Characteristic Curves of Models for ASD Symptom Severity Screening (ADOS-2 and SRS-2) **eMethods 2.** Detailed Information on the Assessment of the ADOS-2 and FSIQ **eMethods 3.** Detailed Explanation of the Training Process **eTable 1.** Model Performances for Screening ASD and Symptom Severity for Each Investigated Split Ratio (Training Set:Test Set) **eTable 2.** Results of Sequential Age-Based Modeling to Screen for ASD

Supplement 2.
Data Sharing Statement
