# Supplementary material for: Development of Deep Ensembles to Screen for Autism and Symptom Severity Using Retinal Photographs
Source: JAMA Netw Open. 2023 Dec 15;6(12):e2347692. doi: 10.1001/jamanetworkopen.2023.47692 (PMC10724768; doi:10.1001/jamanetworkopen.2023.47692)
Supplement: Supplement 1. — eMethods 1. Detailed Description of the Retinal Imaging Environment eFigure 1. Participant Flow Diagram eFigure 2. Receiver Operating Characteristic Curves of Models for ASD Symptom Severity Screening (ADOS-2 and SRS-2) eMethods 2. Detailed Information on the Assessment of the ADOS-2 and FSIQ eMethods 3. Detailed Explanation of the Training Process eTable 1. Model Performances for Screening ASD and Symptom Severity for Each Investigated Split Ratio (Training Set:Test Set) eTable 2. Results of Sequential Age-Based Modeling to Screen for ASD [file jamanetwopen-e2347692-s001.pdf]

Supplementary Online Content

Kim JH, Hong JS, Choi H, et al. Development of deep ensembles to screen for autism and symptom severity using retinal photographs. *JAMA Netw Open*. 2023;6(12):e2347692. doi:10.1001/jamanetworkopen.2023.47692

eMethods 1. Detailed Description of the Retinal Imaging Environment

eFigure 1. Participant Flow Diagram

eFigure 2. Receiver Operating Characteristic Curves of Models for ASD Symptom Severity Screening (ADOS-2 and SRS-2)

eMethods 2. Detailed Information on the Assessment of the ADOS-2 and FSIQ

eMethods 3. Detailed Explanation of the Training Process

eTable 1. Model Performances for Screening ASD and Symptom Severity for Each Investigated Split Ratio (Training Set:Test Set)

eTable 2. Results of Sequential Age-Based Modeling to Screen for ASD

This supplementary material has been provided by the authors to give readers additional information about their work.

© 2023 Kim JH et al. *JAMA Network Open.*

eMethods 1. Detailed Description of the Retinal Imaging Environment

1. Participant recruitment

Participants with autism spectrum disorder (ASD) were expeditiously recruited for the Open AI Dataset Project funded by the Korean government. Two child and adolescent psychiatrists independently confirmed the diagnosis of ASD via semi-structured interviews based on the fifth edition of the Diagnostic and Statistical Manual of Mental Disorders (DSM-5) and the second edition of the Autism Diagnostic Observation Schedule (ADOS-2).

2. Retinal imaging environment

When obtaining retinal photographs of patients with ASD, caregivers accompanied them to ensure comfort and stability. The photography sessions for patients with ASD took place in a space dedicated to their needs, distinct from a general ophthalmology examination room. This space was designed to be warm and welcoming, thus creating a familiar environment for patients. Retinal photographs of typically developing (TD) individuals were obtained in a general ophthalmology examination room.

Each eye required an average of 10–30 s for photography, although some cases involved longer periods to help the patient calm down, sometimes exceeding 5–10 min. All images were captured in a dark room to optimize their quality. Retinal photographs of both patients with ASD and TD were obtained using non-mydriatic fundus cameras, including EIDON (iCare), Nonmyd 7 (Kowa), TRC-NW8 (Topcon), and Visucam NM/FA (Carl Zeiss Meditec).

© 2023 Kim JH et al. *JAMA Network Open.*

eFigure 1. Participant Flow Diagram

Abbreviations: ADOS-2=the second edition of the Autism Diagnostic Observation Schedule, ASD=autism spectrum disorder, DSM-5=the fifth edition of the Diagnostic and Statistical Manual of Mental Disorders, N=number of images, SRS-2=the second edition of the Social Responsiveness Scale, TD=typically developing individuals.

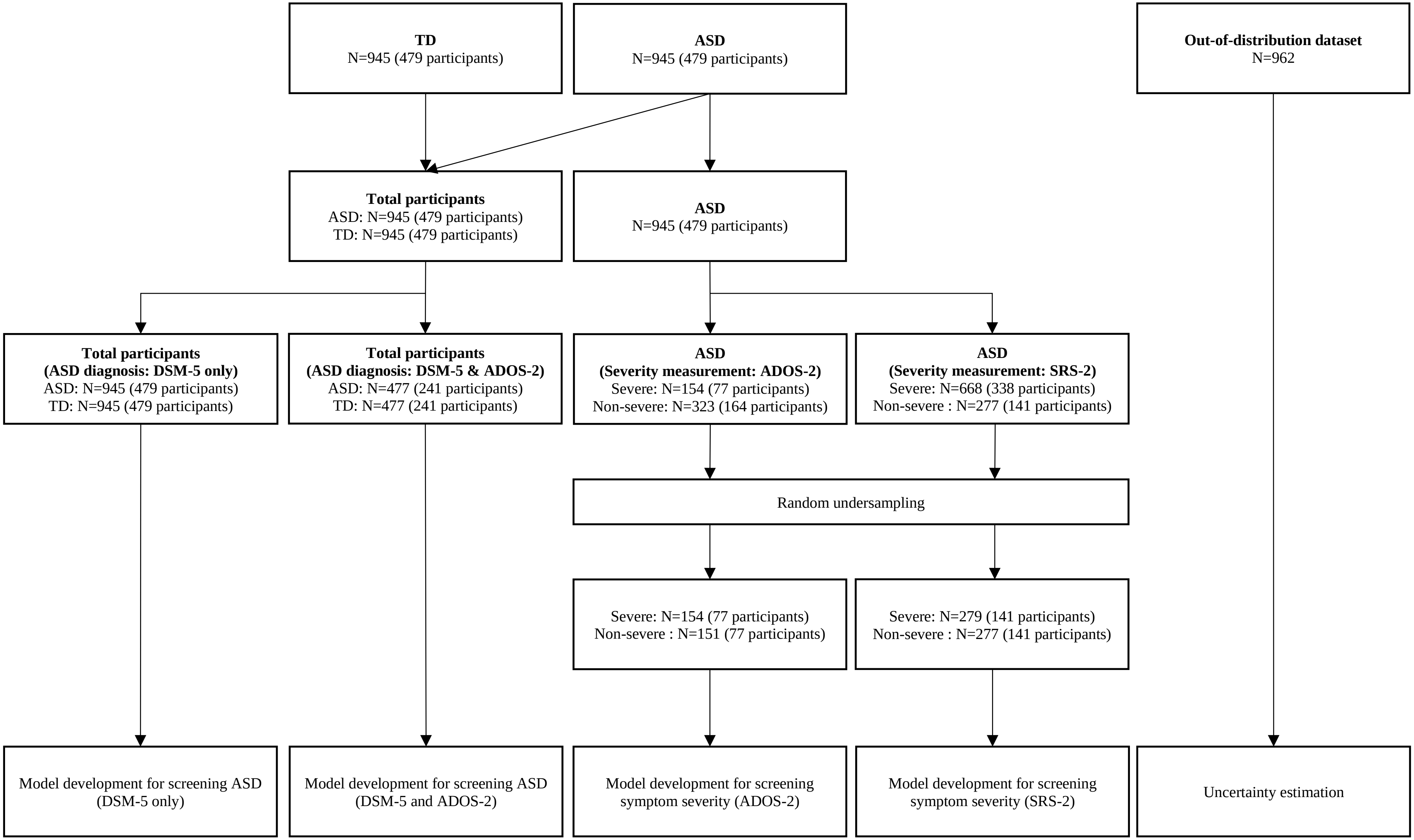

© 2023 Kim JH et al. *JAMA Network Open.*

eFigure 2. Receiver Operating Characteristic Curves of Models for ASD Symptom Severity Screening (ADOS-2 and SRS-2)

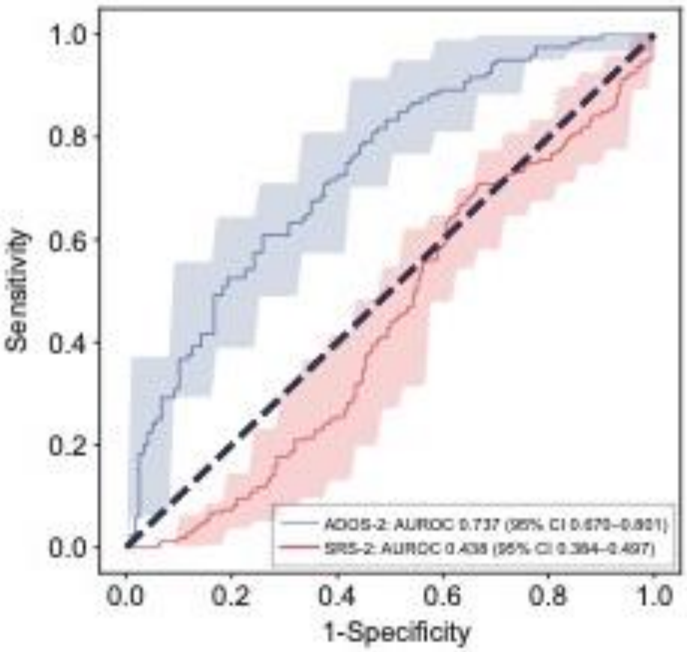

Note: Shaded areas indicate the 95% CIs.

Abbreviations: ADOS-2=Autism Diagnostic Observation Schedule-2, ASD=autism spectrum disorder, AUROC=area under the receiver operating characteristics, CI=confidence interval, SRS-2=Social Responsiveness Scale-2.

© 2023 Kim JH et al. *JAMA Network Open.*

eMethods 2. Detailed Information on the Assessment of the ADOS-2 and FSIQ

1. ADOS-2

In this study, the ADOS examination was conducted by a single examiner who, as an ADOS/ADI-R researcher, had completed the ADOS-2 advanced/research reliability training. Modules T, 1, 2, and 3 were used in the study. The selection of ADOS modules was tailored to each patient's age and language abilities, which trained ADOS Examiners determined.

2. FSIQ

To evaluate the FSIQ of patients with ASD, we employed the K-WPPSI-IV for participants younger than 6 years, the K-WISC-IV for those between 6 and 16 years, and the K-WAIS-IV for individuals aged 17 years or older.

Abbreviations:
K-WPPSI-IV= Korean-Wechsler Preschool and Primary Scale of Intelligence-Fourth Edition,
K-WISC-IV=Korean-Wechsler Intelligence Scale for Children-Fourth Edition
K-WAIS-IV=Korean-Wechsler Adult Intelligence Scale-Fourth Edition

© 2023 Kim JH et al. *JAMA Network Open.*

eMethods 3. Detailed Explanation of the Training Process

We adopted transfer learning using weights from a model trained using ImageNet. Because the last fully connected layer of the pretrained ResNeXt-50 contained 1000 output units, we modified it to have two output units for binary classification. We added a dropout layer after the pooling layer with a probability of 0.5 to avoid possible overfitting. We trained five models in parallel to build deep ensembles because this number was recommended for the robust estimation of predictive uncertainty.[1] The prediction for each image was made by averaging the outputs of the five models (see the diagram below). Finally, a prediction was made at the patient level by averaging the outputs of the two eyes. When a patient had a single image, the prediction was based on the output of one eye (eFigure 1 in Supplement).

We used the binary cross-entropy loss function as a proper scoring rule for the binary classification.[1] The training was performed in batches of 32 images over 100 epochs with an initial learning rate 1e-3. For the learning rate scheduler, we used ReduceLROnPlateau with a patience of 3 and a learning rate decrease factor of 0.1. To prevent overfitting, early stopping criteria were employed by terminating the training process when the validation loss of the tuning set was not reduced for ten epochs. We used an Adam optimizer with a weight decay of 1e-6. During training, general data transformation techniques were applied to each batch for data augmentation, including random vertical flips, horizontal flips, and rotations to address possible overfitting.

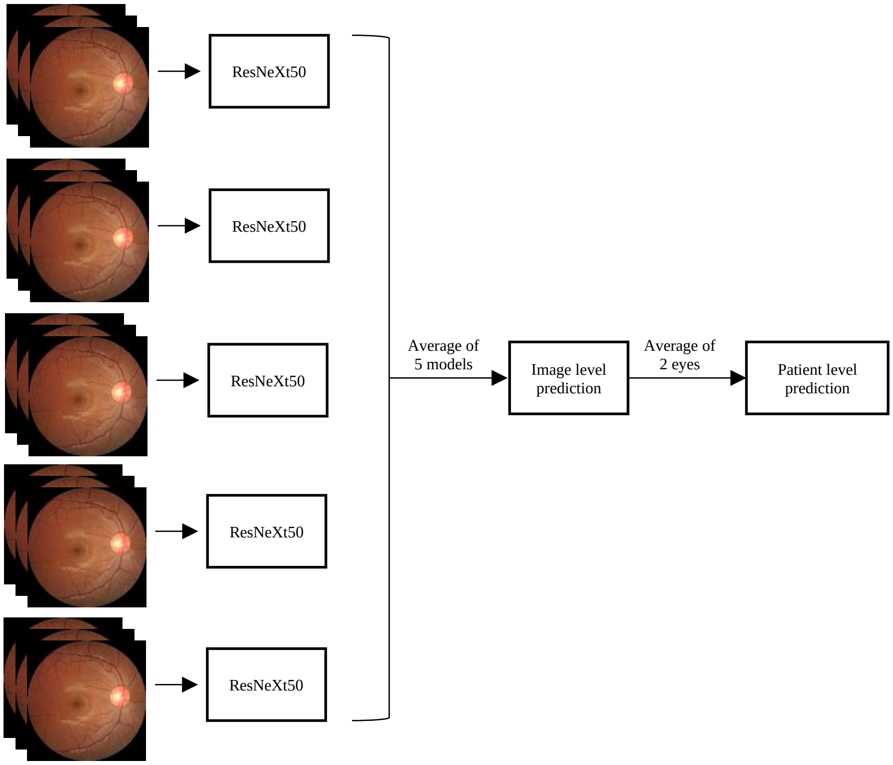

1. Lakshminarayanan B, Pritzel A, Blundell C. Simple and scalable predictive uncertainty estimation using deep ensembles. *Advances in neural information processing systems* 2017; **30**.

© 2023 Kim JH et al. *JAMA Network Open.*

eTable 1. Model Performances for Screening ASD and Symptom Severity for Each Investigated Split Ratio (Training Set:Test Set)

			Classification performance				Calibration performance	
			AUROC (95% CI)	Sensitivity (95% CI)	Specificity (95% CI)	Accuracy (95% CI)	NLL (95% CI)	Brier score (95% CI)
ASD[a] vs. TD	80:20	Single model	1.000 (1.000-1.000)	1.000 (1.000-1.000)	1.000 (1.000-1.000)	1.000 (1.000-1.000)	0.000 (0.000-0.000)	0.000 (0.000-0.000)
		Deep ensemble	1.000 (1.000-1.000)	1.000 (1.000-1.000)	1.000 (1.000-1.000)	1.000 (1.000-1.000)	0.000 (0.000-0.000)	0.000 (0.000-0.000)
	85:15	Single model	1.000 (1.000-1.000)	1.000 (1.000-1.000)	1.000 (1.000-1.000)	1.000 (1.000-1.000)	0.000 (0.000-0.000)	0.000 (0.000-0.000)
		Deep ensemble	1.000 (1.000-1.000)	1.000 (1.000-1.000)	1.000 (1.000-1.000)	1.000 (1.000-1.000)	0.000 (0.000-0.000)	0.000 (0.000-0.000)
	90:10	Single model	1.000 (1.000-1.000)	1.000 (1.000-1.000)	1.000 (1.000-1.000)	1.000 (1.000-1.000)	0.000 (0.000-0.000)	0.000 (0.000-0.000)
		Deep ensemble	1.000 (1.000-1.000)	1.000 (1.000-1.000)	1.000 (1.000-1.000)	1.000 (1.000-1.000)	0.000 (0.000-0.000)	0.000 (0.000-0.000)
ASD[b] vs. TD	80:20	Single model	1.000 (1.000-1.000)	1.000 (1.000-1.000)	1.000 (1.000-1.000)	1.000 (1.000-1.000)	0.000 (0.000-0.000)	0.000 (0.000-0.000)
		Deep ensemble	1.000 (1.000-1.000)	1.000 (1.000-1.000)	1.000 (1.000-1.000)	1.000 (1.000-1.000)	0.000 (0.000-0.000)	0.000 (0.000-0.000)
	85:15	Single model	1.000 (1.000-1.000)	1.000 (1.000-1.000)	1.000 (1.000-1.000)	0.999 (0.999-1.000)	0.020 (0.000-0.050)	0.001 (0.000-0.001)
		Deep ensemble	1.000 (1.000-1.000)	1.000 (1.000-1.000)	1.000 (1.000-1.000)	1.000 (1.000-1.000)	0.000 (0.000-0.000)	0.000 (0.000-0.000)
	90:10	Single model	1.000 (1.000-1.000)	1.000 (1.000-1.000)	0.992 (0.976-1.008)	0.996 (0.988-1.004)	0.138 (0.000-0.419)	0.004 (0.000-0.013)
		Deep ensemble	1.000 (1.000-1.000)	1.000 (1.000-1.000)	1.000 (1.000-1.000)	1.000 (1.000-1.000)	0.000 (0.000-0.000)	0.000 (0.000-0.000)
ADOS-2 (≥ 8 vs < 8)	80:20	Single model	0.636 (0.433-0.839)	0.665 (0.503-0.828)	0.651 (0.454-0.828)	0.679 (0.482-0.876)	11.609 (5.922-1.000)	0.335 (0.172-0.497)
		Deep ensemble	0.709 (0.521-0.896)	0.709 (0.540-0.878)	0.940 (0.825-1.000)	0.469 (0.218-0.720)	9.982 (4.391-1.000)	0.291 (0.127-0.456)
	85:15	Single model	0.673 (0.643-0.704)	0.576 (0.537-0.616)	0.671 (0.630-0.710)	0.624 (0.597-0.651)	12.99 (12.09-13.93)	0.376 (0.350-0.403)
		Deep ensemble	0.737 (0.670-0.801)	0.575 (0.491-0.661)	0.744 (0.670-0.820)	0.659 (0.600-0.725)	11.76 (9.50-13.82)	0.341 (0.275-0.400)
	90:10	Single model	0.670 (0.378-0.957)	0.724 (0.502-0.947)	0.598 (0.286-0.879)	0.849 (0.670-0.994)	9.567 (2.055-1.000)	0.274 (0.058-0.490)
		Deep ensemble	0.793 (0.553-1.000)	0.814 (0.628-0.999)	0.882 (0.645-1.000)	0.738 (0.429-1.000)	6.515 (0.000-1.000)	0.189 (0.009-0.369)
SRS-2 (≥ 76 vs < 76)	80:20	Single model	0.522 (0.371-0.673)	0.568 (0.441-0.695)	0.551 (0.383-0.719)	0.586 (0.422-0.746)	14.877 (10.449-1.000)	0.433 (0.301-0.565)
		Deep ensemble	0.522 (0.369-0.675)	0.576 (0.449-0.704)	0.897 (0.783-1.000)	0.250 (0.091-0.409)	14.545 (10.039-1.000)	0.420 (0.296-0.545)
	85:15	Single model	0.457 (0.433-0.483)	0.497 (0.467-0.527)	0.450 (0.420-0.480)	0.474 (0.452-0.495)	18.17 (17.45-18.96)	0.526 (0.505-0.549)
		Deep ensemble	0.438 (0.384-0.497)	0.523 (0.458-0.594)	0.443 (0.380-0.510)	0.484 (0.440-0.528)	17.83 (16.31-19.44)	0.516 (0.472-0.563)
	90:10	Single model	0.508 (0.290-0.727)	0.593 (0.417-0.769)	0.558 (0.327-0.780)	0.631 (0.430-0.833)	14.019 (7.949-1.000)	0.408 (0.229-0.586)
		Deep ensemble	0.487 (0.259-0.715)	0.623 (0.451-0.796)	0.737 (0.515-0.958)	0.503 (0.234-0.772)	13.129 (7.174-1.000)	0.379 (0.206-0.552)

a. The diagnosis of ASD was based on DSM-5 only.
b. The diagnosis of ASD was based on both DSM-5 and ADOS-2.
Abbreviations: ADOS-2=calibrated severity score of the second edition of the Autism Diagnostic Observation Schedule, ASD=autism spectrum disorder, AUROC=area under the receiver operating characteristics, CI=confidence interval, DSM-5=the fifth edition of the Diagnostic and Statistical Manual of Mental Disorders, NLL=negative log-likelihood, SRS-2=T-score of the second edition of the Social Responsiveness Scale, TD=typically developing individuals

© 2023 Kim JH et al. *JAMA Network Open.*

eTable 2. Results of Sequential Age-Based Modeling to Screen for ASD

We reconstructed models for screening for autism spectrum disorder (ASD) by considering age, starting from the youngest age within our sample and progressing cumulatively. To do this, we generated new models by training them with data from those aged 4 (as four was the youngest age in our sample) and assessed their performance using this specific dataset. We repeated this process by building models using data from individuals aged 5 years and younger then, from those aged 6 years and younger.

		No. of participants	No. of images	AUROC (95% CI)	Sensitivity (95% CI)	Specificity (95% CI)	Accuracy (95% CI)
Cut-off age	4	82	152	1.000 (1.000-1.000)	1.000 (1.000-1.000)	1.000 (1.000-1.000)	1.000 (1.000-1.000)
	≤5	232	446	1.000 (1.000-1.000)	1.000 (1.000-1.000)	1.000 (1.000-1.000)	1.000 (1.000-1.000)
	≤6	456	888	1.000 (1.000-1.000)	1.000 (1.000-1.000)	1.000 (1.000-1.000)	1.000 (1.000-1.000)
	≤7	570	1116	1.000 (1.000-1.000)	1.000 (1.000-1.000)	1.000 (1.000-1.000)	1.000 (1.000-1.000)
	≤8	654	1282	1.000 (1.000-1.000)	1.000 (1.000-1.000)	1.000 (1.000-1.000)	1.000 (1.000-1.000)
	≤9	722	1418	1.000 (1.000-1.000)	1.000 (1.000-1.000)	1.000 (1.000-1.000)	1.000 (1.000-1.000)
	≤10	760	1494	1.000 (1.000-1.000)	1.000 (1.000-1.000)	1.000 (1.000-1.000)	1.000 (1.000-1.000)
	≤11	820	1614	1.000 (1.000-1.000)	1.000 (1.000-1.000)	1.000 (1.000-1.000)	1.000 (1.000-1.000)
	≤12	850	1674	1.000 (1.000-1.000)	1.000 (1.000-1.000)	1.000 (1.000-1.000)	1.000 (1.000-1.000)
	≤13	880	1734	1.000 (1.000-1.000)	1.000 (1.000-1.000)	1.000 (1.000-1.000)	1.000 (1.000-1.000)
	≤14	922	1818	1.000 (1.000-1.000)	1.000 (1.000-1.000)	1.000 (1.000-1.000)	1.000 (1.000-1.000)
	≤15	934	1842	1.000 (1.000-1.000)	1.000 (1.000-1.000)	1.000 (1.000-1.000)	1.000 (1.000-1.000)
	≤16	938	1850	1.000 (1.000-1.000)	1.000 (1.000-1.000)	1.000 (1.000-1.000)	1.000 (1.000-1.000)
	≤17	944	1862	1.000 (1.000-1.000)	1.000 (1.000-1.000)	1.000 (1.000-1.000)	1.000 (1.000-1.000)
	≤18	958	1890	1.000 (1.000-1.000)	1.000 (1.000-1.000)	1.000 (1.000-1.000)	1.000 (1.000-1.000)

Abbreviations: AUROC= area under the receiver operating characteristics, CI=confidence interval

© 2023 Kim JH et al. *JAMA Network Open.*
