# Supplementary material for: Development of Deep Ensembles to Screen for Autism and Symptom Severity Using Retinal Photographs
Source: JAMA Netw Open. 2023 Dec 15;6(12):e2347692. doi: 10.1001/jamanetworkopen.2023.47692 (PMC10724768; doi:10.1001/jamanetworkopen.2023.47692)
Supplement: Supplement 2. — Data Sharing Statement [file jamanetwopen-e2347692-s002.pdf]

Data Sharing Statement

Kim. Development of Deep Ensembles to Screen for Autism and Symptom Severity Using Retinal Photographs. *JAMA Netw Open*. Published December 15, 2023. doi:10.1001/jamanetworkopen.2023.47692

Data
Data available: Yes
Data types: Deidentified participant data
How to access data: The study data can be shared by contacting the corresponding authors (YRP: yurangpark@yuhs.ac or K-AC: kacheon@yuhs.ac) upon reasonable request.
When available: With publication

Supporting Documents
Document types: Statistical/analytic code
How to access documents: The code supporting the findings of this study is openly available at: https://github.com/DigitalHealthcareLab/22ASDRetina.
When available: beginning date: 05-21-2023

Additional Information
Who can access the data: Data used in this study will be available to anyone requesting the data to the corresponding authors (YRP: yurangpark@yuhs.ac or K-AC: kacheon@yuhs.ac) with a reasonable request
Types of analyses: for any purpose
Mechanisms of data availability: with a signed data access agreement
